# Supplementary material for: Intra-atrial activation pattern is useful to localize the areas of non-pulmonary vein triggers of atrial fibrillation
Source: PLoS One. 2022 Apr 25;17(4):e0264894. doi: 10.1371/journal.pone.0264894 (PMC9037930; doi:10.1371/journal.pone.0264894)
Supplement: Attachment — Submitted filename: renamed_2ca88.docx [file pone.0264894.s002.docx]

Reviewer #1: Point two on the role of extrapulmonary substrates has not been adequately investigated. I believe it is not possible to treat intra-atrial delays without talking about fibrosis!

Thank you for your excellent comment again about the atrial fibrosis.　To address the importance of the relationship between left atrial degeneration and arrhythmogenic substrate in AF ablation, the following sentence and the reference below were added in Discussion and Reference.

*(Discussion)*

*For detailed mapping, it is also important to evaluate the substrate such as fibrosis by using multipolar electrodes [22].*

*(Reference)*

*22. Rillo M, Palamà Z, Punzi R, Vitanza S, Aloisio A, Polini S, et al. A new interpretation of nonpulmonary vein substrates of the left atrium in patients with atrial fibrillation. J Arrhythm. 2021;37:338-47.*
